# 浆膜腔积液细胞病理学国际报告系统在肺癌相关浆膜腔积液分层诊断中的临床应用研究

**DOI:** 10.3779/j.issn.1009-3419.2026.102.03

**Published:** 2026-01-20

**Authors:** Wei WU, Jian ZHANG, Cong WANG, Xinxiang CHANG, Yue SUN, Linlin ZHAO, Shize WANG, Yiyun ZHANG, Huan ZHAO, Huiqin GUO, Zhihui ZHANG

**Affiliations:** ^1^030012 太原，山西省人民医院检验科（武炜）; ^1^Department of Laboratory Medicine, Shanxi Provincial People’s Hospital, Taiyuan 030012, China; ^2^100021 北京，国家癌症中心，国家肿瘤临床医学研究中心，中国医学科学院北京协和医学院肿瘤医院病理科细胞学室（武炜，张健，王聪，常馨香，孙悦，赵琳琳，王世泽，张译允，赵焕，郭会芹，张智慧）; ^2^Section of Cytopathology, Department of Pathology, National Cancer Center/National Clinical Research Center for Cancer/Cancer Hospital, Chinese Academy of Medical Sciences and Peking Union Medical College, Beijing 100021, China

**Keywords:** 肺肿瘤, 浆膜腔积液, 浆膜腔积液细胞病理学国际报告系统, 免疫细胞化学, 分层诊断, Lung neoplasms, Serous effusion, The International System for Serous Fluid Cytopathology, Immunocytochemistry, Stratified diagnosis

## Abstract

**背景与目的:**

浆膜腔积液是中晚期肺癌患者常见并发症，其出现往往提示肿瘤已发生胸膜或其他浆膜转移。准确诊断积液中的肿瘤细胞并进行病理分型，对指导临床治疗至关重要。然而，单纯依赖细胞形态学诊断存在局限性，尤其在不典型病例中易出现不确定性诊断。《浆膜腔积液细胞病理学国际报告系统》（The International System for Serous Fluid Cytopathology, TIS）提出了形态学与辅助技术相结合的标准化分层诊断框架，但其在国内肺癌人群中的临床应用价值尚缺乏大规模验证。本研究旨在基于大样本肺癌病例，系统性评估TIS系统在肺癌浆膜腔积液诊断与分型中的效能。

**方法:**

回顾性纳入2018年1月至2023年12月中国医学科学院肿瘤医院的1274例浆膜腔积液样本，其均来源于组织病理学和/或临床病史确诊的肺癌患者。严格遵循TIS系统诊断流程，先进行形态学评估并分级（I-V级），对部分形态学不确定（III、IV级）及V级病例，利用细胞蜡块进行免疫细胞化学（immunocytochemistry, ICC）检测，综合确定最终诊断级别并进行肿瘤分型与起源判断。统计分析形态学初诊与联合ICC诊断一致性、分级变化趋势及ICC检测在病理分型与溯源中的临床价值。

**结果:**

形态学初诊中，83.1%（1059/1274）的病例直接诊断为恶性（V级），12.3%（157/1274）的病例为不确定性诊断（III或IV级）。对69例接受ICC检测的病例进一步分析显示，升级诊断率为85.5%（59/69），其中IV级病例的升级比例（89.6%, 43/48）高于III级（76.2%, 16/21）；7.2%（5/69）的III级病例降级为良性（II级），另7.2%（5/69）因细胞量不足或分化差而维持原级，表明ICC可显著提升肺癌浆膜腔积液不确定性诊断的明确性。在最终诊断恶性的病例中，ICC对542例进行分型与溯源，533例（98.3%）实现精准病理分型（腺癌86.7%、小细胞癌4.4%、鳞癌3.9%），510例（94.0%）明确为肺来源。关键ICC标志物表达谱分析显示，浆膜腔积液中肺腺癌甲状腺转录因子-1（thyroid transcription factor-1, TTF-1）与Napsin A呈高表达，阳性率分别为93.0%和76.2%；肺鳞癌特征性表达P40和P63，阳性率分别为60.0%和73.7%；小细胞癌强表达Syn与CD56，阳性率分别达87.0%和81.8%。通过采用一组互补抗体进行综合判读，可以对形态学初诊报告进行验证与补充，为亚型鉴别提供可靠依据。

**结论:**

TIS系统构建的“形态学初诊、辅助技术确证”分层诊断路径，能显著提高肺癌浆膜腔积液诊断的准确性，有效优化不确定性病例分流，实现高精度的病理分型与组织起源判断。建议在临床实践中推广应用TIS系统，以促进浆膜腔积液细胞病理学诊断的规范化与精准化。

## Abstract (en)

**Background and objective:**

Serous effusion is a common complication in patients with advanced lung cancer, which often indicates that the tumor has metastasized to the pleura or other serosal membranes. It is essential to achieve an accurate diagnosis and pathological classification of tumor cells in effusion to guide clinical treatment. However, there are limitations to relying solely on morphological diagnosis, especially in atypical cases where diagnostic uncertainty is common. The International System for Serous Fluid Cytopathology (TIS) proposes a standardized hierarchical diagnostic framework that integrates morphology and auxiliary techniques. Nevertheless, its clinical application value in the Chinese lung cancer population remains insufficiently validated. This study aimed to systematically evaluate the diagnostic performance of the TIS system in the assessment and classification of serous effusions based on a large sample of lung cancer cases.

**Methods:**

A retrospective analysis was conducted on 1274 serous effusion specimens from the Cancer Hospital Chinese Academy of Medical Sciences between January 2018 and December 2023, all of which were derived from patients with lung cancer confirmed by histopathology and/or clinical history. The diagnostic procedure strictly followed the TIS protocol, which commenced with the morphological evaluation and assignment to categories I-V. For some cases with ambiguous morphology (categories III and IV) and category V, immunocytochemistry (ICC) was performed using cell blocks to comprehensively determine the final diagnostic category, tumor subtyping, and tumor origin. Statistical analyses were applied to assess the diagnostic concordance between initial morphology and combined ICC. Additionally, the trend in category changes and the clinical value of ICC in pathological subtyping and origin determination were analyzed.

**Results:**

Based on morphology alone, 83.1% (1059/1274) of cases were classified as malignant (category V), while 12.3% (157/1274) were categorized as indeterminate (categories III or IV). Further analysis of the 69 cases that underwent ICC detection revealed that the rate of upgraded diagnosis was 85.5% (59/69), and the upgrading proportion in category IV cases (89.6%, 43/48) was significantly higher than that in category III cases (76.2%, 16/21). Furthermore, 7.2% (5/69) of category III cases were downgraded to benign (category II), while another 7.2% (5/69) remained unchanged due to insufficient cellularity or poor differentiation. These findings indicate that ICC can markedly improve the diagnostic accuracy for indeterminate serous effusions in lung cancer. Among definitively malignant cases, ICC was employed to enable subtyping and origin assessment in 542 cases, achieving precise pathological classification in 533 cases (98.3%): including adenocarcinoma (86.7%), small cell carcinoma (4.4%), and squamous cell carcinoma (3.9%), and confirming pulmonary origin in 510 cases (94.0%). Analysis of key ICC marker expression profiles revealed that lung adenocarcinoma in serous effusions showed high expressions of thyroid transcription factor-1 (TTF-1) and Napsin A (positivity rates: 93.0% and 76.2%, respectively); lung squamous cell carcinoma characteristically expressed P40 and P63 (positivity rates: 60.0% and 73.7%, respectively); and small cell carcinoma strongly expressed Syn and CD56 (positivity rates: 87.0% and 81.8%, respectively). Integrated interpretation using a complementary antibody panel effectively validated and supplemented morphological findings, providing a reliable basis for subtype differentiation.

**Conclusion:**

The TIS-based stratified diagnostic pathway of “initial morphological diagnosis followed by ancillary techniques confirmation” significantly improves diagnostic accuracy for lung cancer-associated serous effusions, optimizes the management of indeterminate cases, and achieves high-precision pathological subtyping and determination of tumor origin. It is recommended to widely implement the TIS system in clinical practice to enhance standardization and diagnostic precision in serous fluid cytopathology.

国际上肺癌的发病率和死亡率均居恶性肿瘤首位^[1]^。中国肺癌的疾病负担情况同样严峻。国家癌症中心发布的《2022年中国癌症发病率和死亡率》报告^[2]^显示，肺癌新发病例约106.06万，占全部恶性肿瘤发病数的22.0%；死亡病例约73.33万，占全部恶性肿瘤死亡数的28.5%，其发病率和死亡率均位居我国恶性肿瘤之首，对居民健康造成严重影响。

浆膜腔积液（包括胸腔积液、腹腔积液及心包积液等）是中晚期肺癌患者常见的并发症，尤以恶性胸腔积液最为多见。文献^[3]^报道约15%的肺癌患者初诊时即合并胸腔积液，50%的患者疾病进展过程中发展为恶性胸腔积液。积液的形成提示肿瘤已经发生胸膜或其他浆膜转移。由于不同组织学类型（如腺癌、鳞癌、小细胞癌等）的肺癌，其系统性治疗策略（包括化疗、靶向及免疫治疗）存在显著差异^[4-6]^，因此在开始治疗前，对肺癌细胞进行精准病理学分型，已成为临床制定个体化治疗方案的关键前提。

然而，对于伴发浆膜腔积液的晚期肺癌患者，不适宜手术且经常难以获取组织活检标本，其病理学诊断很大程度依赖浆膜腔积液细胞学检查结果，包括传统涂片与细胞蜡块技术等。为提高浆膜腔积液细胞病理学诊断的标准化与可重复性，2023年由国内学者引进国际细胞学会出版的《浆膜腔积液细胞病理学国际报告系统》（The International System for Serous Fluid Cytopathology, TIS）^[7]^。该系统构建了以形态学为基础、整合辅助技术的分层诊断框架，显著提升了诊断的准确性，具有临床指导价值。但是，该报告系统在国内临床应用中的实际效能尚缺乏大规模样本研究的验证与报道。

本研究拟纳入经临床已经证实为肺癌患者的浆膜腔积液样本，依据上述TIS系统进行形态学分级与免疫细胞化学（immunocytochemistry, ICC）辅助检测，系统评估其在肺癌浆膜腔积液诊断与分型中的应用价值，以期为我国肺癌浆膜腔积液的规范化细胞病理诊断提供数据支持与实践参考。

## 1 资料与方法

### 1.1 研究对象

本研究为一项回顾性研究。研究对象为2018年1月至2023年12月于中国医学科学院肿瘤医院就诊的1274例肺癌患者，其中542例签署知情同意书并进行ICC检测。病例纳入标准：（1）原发灶经手术/活检的组织病理学和/或临床病史确诊为肺癌；（2）所有浆膜腔积液标本，均有依据TIS报告系统进行细胞病理学诊断的明确分级结果。

### 1.2 患者临床资料

1274例患者中位年龄为62岁，年龄范围为27-96岁。男性679例（53.3%），女性595例（46.7%）；浆膜腔积液样本中1184例（93.0%）为胸腔积液，35例（2.7%）为腹腔积液，55例（4.3%）为心包积液。原发肿瘤的组织病理类型为腺癌1065例（83.6%），鳞癌46例（3.6%），小细胞癌50例（3.9%），其他46例（3.6%），未明确类型67例（5.3%）。

### 1.3 细胞病理学诊断分级

所有浆膜腔积液标本的细胞病理学诊断均严格依据TIS报告系统进行分级。具体诊断流程见图1。首先，每例标本均制备传统涂片、液基薄片和细胞蜡块，染色后由2名高年资细胞病理医师采用双盲法独立阅片。依据该系统定义的细胞形态学标准，将病例初步分为5个诊断级别：I级（无法诊断）、II级（未见恶性）、III级（意义不明确的非典型性）、IV级（可疑恶性）以及V级（恶性）。若初步诊断意见存在分歧，则通过共同复阅、参考临床病史和影像学资料进行讨论，直至达成一致共识，形成基于形态学的初步诊断报告。其次，对部分形态学诊断为III、IV级的病例，以及形态学诊断为V级且需要进一步分型或溯源的病例，采用细胞蜡块进行ICC检测。最后，综合细胞形态学与ICC结果确定最终诊断分级（I-V级）。依据最终分级结果进行后续的数据分析，以评估该系统在鉴别诊断、降低诊断不确定性及明确肿瘤分型方面的临床应用价值。

**图1:**
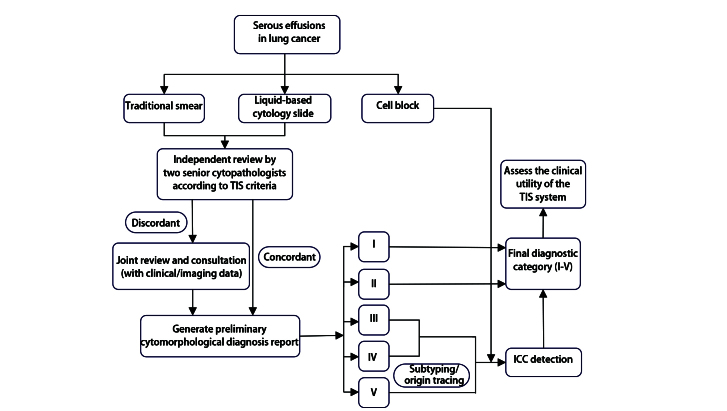
肺癌患者浆膜腔积液标本细胞病理学诊断流程图

### 1.4 传统涂片和液基薄片的制备

传统细胞学涂片制备：送检的浆膜腔积液标本要求至少75 mL，将液体充分混匀后置于离心管中，以2500 rpm离心10 min。弃上清，选取灰白色细胞层，匀速推片，制备传统涂片1张（长度2-4 cm）。根据沉淀物黏稠度调整推片速度。涂片经95%乙醇固定15 min后，进行苏木素-伊红（hematoxylin-eosin, HE）染色。液基薄层细胞学制片：采用新柏氏液基细胞学技术（ThinPrep®, Hologic, USA）进行制片。步骤如下：（1）浆膜腔积液离心弃上清后，选取灰白色细胞层，置入ThinPrep消化液中，振荡10 min后离心，将细胞转入保存液；（2）薄层制片：经处理的细胞在负压作用下吸附于滤膜表面，形成单层细胞层，随后被直接转移至带正电荷的载玻片上，形成直径20 mm薄层细胞片；（3）固定与染色：涂片经95%乙醇固定后，采用巴氏染色法进行染色。

### 1.5 细胞蜡块制备

浆膜腔积液离心弃上清后，选取灰白色细胞层置于1.5 mL EP管中，加入0.5 mL抗凝血浆混匀，再加入0.5 mL（100 U/mL）血浆凝固酶，混匀，静置1 min，将沉淀物用滤纸包裹，中性甲醛溶液固定至少2 h，以防止细胞自溶。依次经梯度乙醇脱水、二甲苯透明、石蜡浸渍。最后进行石蜡包埋，制成细胞蜡块。所有蜡块均连续切片，切片厚度为4 μm，用于后续ICC检测。

### 1.6 ICC染色及结果判读 应用罗氏BenchMark

GX自动染色仪对浆膜腔积液标本的细胞蜡块进行ICC染色，检测甲状腺转录因子-1（thyroid transcription factor-1, TTF-1）（克隆号：OTI1E3）、Napsin A（MX015）、CK7（UMAB161）、EA（Ber-EP4）、Moc-31（MOC-31）、P40（MXR010）、P63（MX013）、CK5/6（MX040）、Syn（MX038）、CD56（MX039）、ChrA（MX018）蛋白表达。染色步骤严格按照仪器标准化染色程序进行。其中，抗TTF-1、CK7抗体购自北京中杉金桥生物技术有限公司，抗Napsin A、Moc-31、P40、P63、Syn、CD56、ChrA、CK5/6抗体购自福州迈新生物技术开发有限公司，抗EA抗体购自基因科技（上海）股份有限公司。依据细胞形态学提示及鉴别诊断需求进行ICC抗体组合选择。

评估整张涂片的ICC染色强度，各指标均由2名高年资细胞病理医师独立判读，意见不一致时通过讨论达成共识。TTF-1、P40、P63定位于细胞核；Napsin A、CK7、CK5/6及ChrA定位于细胞质；Syn呈胞质内颗粒状着色；EA、Moc-31表达于细胞质和/或细胞膜；CD56定位于细胞膜。经DAB显色后，所有阳性信号均呈棕黄色。每批次染色均设置阳性和阴性对照，对照染色符合预期结果时才被采纳。

### 1.7 统计学方法

采用SPSS 27.0软件进行统计学分析。计数资料以例数（%）表示，计量资料以中位数（范围）表示。采用Kappa检验评估形态学初诊与联合ICC后最终诊断之间的一致性强度：Kappa值≤0.20为一致性差，0.21-0.40为一般，0.41-0.60为中等，0.61-0.80为良好，0.81-0.92为极好，0.93-1.00为优秀^[8]^。采用McNemar-Bowker检验评估诊断分级变化的系统性趋势。两组间升级率的比较采用Fisher精确概率法，P<0.05为差异具有统计学意义。

## 2 结果

### 2.1 形态学与联合ICC诊断后肺癌细胞病理学诊断分级变化

依据TIS报告系统，1274例肺癌患者浆膜腔积液标本的细胞病理学诊断分级在形态学初诊与联合ICC检测后发生变化。依据细胞形态学，诊断为V级者1059例（83.1%），IV级109例（8.6%），III级48例（3.7%），II级56例（4.4%），I级2例（0.2%）。经ICC辅助诊断后，最终分级为：V级1116例（87.6%），IV级68例（5.3%），III级27例（2.1%），II级61例（4.8%），I级2例（0.2%）。联合ICC检测后，V级诊断比例提高4.5%，II级比例增加0.4%；而III级比例降低1.6%，IV级比例降低3.3%，不确定性诊断（III与IV级）总比例从12.3%下降至7.4%（表1）。

**表1:** 浆膜腔积液细胞病理形态学初诊与联合ICC诊断分级比较（n=1274）

TIS category	Cytomorphology alone [n (%)]	Cytomorphology with ICC [n (%)]
I (Nondiagnostic)	2 (0.2)	2 (0.2)
II (Negative for malignancy)	56 (4.4)	61 (4.8)
III (Atypia of undetermined significance)	48 (3.7)	27 (2.1)
IV (Suspicious for malignancy)	109 (8.6)	68 (5.3)
V (Malignant)	1059 (83.1)	1116 (87.6)

### 2.2 诊断分级变化趋势与系统性差异分析

评估1274例浆膜腔积液细胞病理学标本在单纯形态学初诊与联合ICC检测后诊断分级的一致性情况（图2A），以确定其具体变化趋势。Kappa一致性检验分析显示，两种方法总体观察一致率为95.0%（1210/1274），加权Kappa值为0.810（95%CI: 0.761-0.852），提示诊断一致性极好（P<0.001）。然而，McNemar-Bowker对称性检验提示分级变化存在显著的非随机性系统性趋势（χ²=64.000, df=4, P<0.001）。具体地，95例最终不确定性诊断（III和IV级）中有69例做ICC检测并进行分流（图2B、2C）。21例III级病例，经ICC检测后均被分流，明确诊断19例（90.5%）：14例（66.7%）升级为V级确诊恶性，5例（23.8%）降级为II级确诊良性；另有2例（9.5%）III级升级为IV级可疑恶性。48例IV级可疑恶性病例经ICC检测后，43例（89.6%）升级为V级确诊恶性，无降级病例。总体来看，57例（82.6%）升级为恶性（V级），2例（2.9%）升级为可疑恶性（IV级），升级诊断率为85.5%（59/69），IV级病例的升级比例（89.6%, 43/48）高于III级（76.2%, 16/21），但差异无统计学意义（P=0.159）。因此，ICC检测可以对形态学不确定性病例进行诊断优化，提高诊断的确定性。

**图2:**
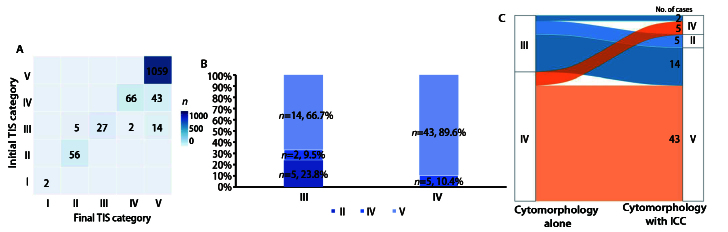
浆膜腔积液细胞病理形态学初诊与联合ICC检测诊断分级变化。A：形态学初诊与联合ICC诊断一致性热图；B：III、IV级病例经ICC后级别变化（n, %）。横坐标为形态学初诊的不确定性诊断分级，纵坐标为病例占比；C：形态学不确定性诊断（Ⅲ和Ⅳ级）经ICC后分流情况。

### 2.3 ICC检测对肺癌浆膜腔积液细胞分型与组织起源的判断价值

在最终诊断为V级的病例中，对542例进行ICC检测以判断肿瘤细胞分型与组织起源。肿瘤细胞分型显示（图3），以腺癌470例（86.7%）为主，其次为小细胞癌24例（4.4%）和鳞癌21例（3.9%），其他类型共18例（3.4%），无法确定类型9例（1.7%）。组织起源分析显示（表2），510例（94.0%）明确为肺来源，包括肺腺癌、鳞癌、小细胞癌及大细胞神经内分泌癌等；仅2例（0.4%）分别判断为卵巢和消化道来源的腺癌；另有30例（5.6%）因细胞量少或标志物表达不典型而无法确定组织起源。表明ICC能对恶性浆膜腔积液实现精确的病理分型，并准确判断肿瘤细胞起源。

**图3:**
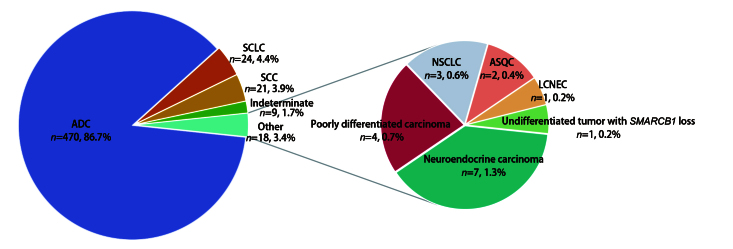
542例浆膜腔积液标本ICC检测后最终细胞病理分型分布

**表2:** 542例浆膜腔积液ICC检测后肿瘤细胞起源分布

Origin tracing	Subtype	n (%)
Lung	ADC, SCC, SCLC, LCNEC, BSCLC	510 (94.0)
Ovary	ADC	1 (0.2)
Gastrointestinal tract	ADC	1 (0.2)
Undetermined	Scant tumor cells	15 (2.8)
	Poorly differentiated cells with atypical immunophenotype	15 (2.8)

### 2.4 肺癌亚型中关键ICC标志物的表达特征分析

对占主导地位的3种肺癌亚型（腺癌、鳞癌与小细胞癌）进行关键ICC标志物分析，其表达特征见表3。腺癌组（n=470）呈现典型的肺腺癌表型：TTF-1与Napsin A呈高表达，阳性率分别为93.0%与76.2%，其中76.0%的病例为TTF-1/Napsin A双阳性模式；而鳞状分化标志物（P40、P63）及神经内分泌标志物（Syn、CD56）多为阴性或低表达。鳞癌组（n=21）特征性表达P40（60.0%）与P63（73.7%），63.2%的病例呈现P40/P63双阳性；而腺癌标志物TTF-1与Napsin A呈低表达或基本不表达（阳性率分别为9.5%和0.0%）。小细胞癌组（n=24）强表达神经内分泌标志物Syn（87.0%）与CD56（81.8%），68.2%为Syn/CD56双阳性；部分病例（71.4%）TTF-1亦呈阳性，而P40与Napsin A均为阴性。上述特征性ICC标志物表达谱为浆膜腔积液肺癌亚型的鉴别诊断提供可靠依据。

**表3:** 肺癌亚型中关键ICC标志物的表达

Pathologic subtype	n	Positive markers	Negative markers	Combination pattern
ADC	470	TTF-1: 93.0% (424/456) Napsin A: 76.2% (333/437) CK7: 97.5% (231/237) EA: 56.3% (152/270) Moc-31: 77.1% (91/118)	P40: 0.7% (2/298) P63: 7.8% (7/90) Syn: 6.8% (5/74) CD56: 15.2% (5/33)	TTF-1+/Napsin A+: 76.0% (330/434)
SCC	21	P40: 60.0% (12/20) P63: 73.7% (14/19) CK5/6: 71.4% (5/7)	TTF-1: 9.5% (2/21) Napsin A: 0.0% (0/19)	P40+/P63+: 63.2% (12/19)
SCLC	24	Syn: 87.0% (20/23) CD56: 81.8% (18/22) ChrA: 50.0% (6/12) EA: 26.7% (4/15) Moc-31: 12.5% (1/8)	P40: 0.0% (0/14) Napsin A: 0.0% (0/14) TTF-1: 71.4% (15/21)	Syn+/CD56+: 68.2% (15/22)

TTF-1: thyroid transcription factor-1.

## 3 讨论

TIS报告系统的建立是细胞病理学诊断标准化进程中的重要里程碑。其核心在于引入一套国际公认的五级诊断分类框架（I-V级），旨在消除诊断术语的歧义，促进实验室间的有效交流。同时，该系统为每个分级明确了恶性风险范围及规范的临床处理建议，提升与临床医师沟通的准确性。TIS报告系统还对近年来逐渐完善的ICC检测和新抗体的使用以及分子诊断技术在浆膜腔积液中的应用进行梳理与阐述，并结合实践经验对原发性与转移性肿瘤的细胞形态学特征进行归纳说明。本研究依托中国医学科学院肿瘤医院1274例肺癌病例，首次在国内以大规模临床样本为基础，系统性地验证其在肺癌浆膜腔积液诊断中的临床应用价值。

基于该系统，首先评估了单纯细胞形态学的诊断效能。形态学初诊直接判定为V级的比例高达83.1%。与既往文献报道相比，综合性医院常规细胞病理学检查所报告的恶性浆膜腔积液检出率为25.2%^[9]^，肿瘤专科医院的检出率增加至47.8%^[10]^，而本研究的结果显著高于上述两类机构，反映了作为国家癌症中心/肿瘤专科医院的样本特征，即诊疗的多为疑难或中晚期转诊病例，肿瘤负荷更高，细胞学阳性检出率也相应提升。同时进一步证明，细胞病理医师只要具备足够的形态学经验，细胞形态学诊断本身就可以达到较高的敏感性与特异性。尽管如此，仍有12.3%病例被归入III级或IV级，凸显了单纯依靠形态学在面对反应性增生、细胞退变或异型性不典型时的局限性。这也印证了TIS系统设立不确定性诊断类别的必要性，为后续辅助检测指明方向。

使用辅助技术ICC检测后，诊断的明确性得到显著改善。形态学上不确定性诊断（III、IV级）病例中，92.8%（64/69）的病例诊断级别发生改变，其中82.6%（57/69）的病例升级为V级，最终确诊为癌；7.2%（5/69）的病例降级为II级，其非典型细胞为增生的间皮细胞；2.9%（2/69）III级病例升级为IV级，虽增高肿瘤风险但未达到明确诊断阈值，证明TIS系统将ICC纳入辅助诊断流程的重要性和前瞻性。然而，仍有7.2%（5/69）的病例维持原诊断级别，ICC未能提供明确的辅助诊断信息。其原因包括：（1）3例（60.0%）因细胞蜡块中肿瘤细胞数量不足，无法满足ICC判读要求；（2）2例（40.0%）由于肿瘤细胞分化差，缺乏特异性ICC标志物表达而呈现不典型表达模式。这些现象揭示了TIS系统在当前技术条件下的诊断边界，也提示未来需从多方面进行技术优化，例如：改进细胞富集技术以提高细胞数量；探索更为灵敏和特异的ICC标志物组合；开发人工智能辅助判读系统提升诊断的客观性和准确性。在部分有肺癌病史的患者中，为确定靶向或免疫治疗的适用性，治疗前常常需要进行基因突变检测。TIS系统明确推荐对浆膜腔积液标本进行分子检测，包括对细胞蜡块进行下一代测序（next-generation sequencing, NGS）或逆转录-聚合酶链式反应（reverse transcription-polymerase chain reaction, RT-PCR）检测，以及对上清液游离DNA（cell-free DNA, cfDNA）等液体标本开展基因突变分析。分子检测不仅可为靶向/免疫治疗提供必要的突变基因信息，也有助于降低诊断中的残余不确定性^[11,12]^。更重要的是，细胞蜡块联合ICC检测能够准确鉴别浆膜腔积液中恶性肿瘤的分型及起源^[13]^。本研究中，在最终诊断为恶性的病例中，基于细胞蜡块进行ICC检测，98.3%（533/542）的病例实现精准病理分型，94.0%（510/542）的病例被确认为肺来源。另外，有2例（0.4%）分别被判定为卵巢和消化道来源的腺癌，这一发现提示，在临床诊断为肺癌的患者中，其浆膜腔积液可能极少数源于第二原发恶性肿瘤的转移，而非原发肺癌。因此，即使患者有明确的肺癌病史，当浆膜腔积液细胞ICC表型与典型肺癌特征不符时，需在鉴别诊断中考虑其他原发灶转移的可能性。本研究充分证明，细胞蜡块能够提供与组织蜡块一致的形态学特征和抗原完整性，这一结论与既往研究^[14-16]^一致。细胞蜡块的制备与应用，是实现浆膜腔积液细胞病理学诊断从常规细胞学判读迈向精准病理学诊断的关键技术环节，也是TIS系统强调的核心步骤。

分析主要肺癌亚型ICC标志物表达谱，发现其在转移性浆膜腔积液中的表达模式与文献中报道的肺癌原发灶样本具有可比性，但也存在一定差异。例如，TTF-1在浆膜腔积液肺来源的腺癌、鳞癌及小细胞癌中的阳性率分别为93.0%、9.5%、71.4%，与其在纤维支气管镜刷检中，原发性肺腺癌（96.0%）、肺鳞癌（8.1%）及肺小细胞癌（85.7%）中的表达趋势基本吻合^[17]^。Napsin A在转移性浆膜腔积液肺腺癌中的阳性率为76.2%，与原发灶数据（82.0%）接近；而P40在转移性浆膜腔积液肺鳞癌中的表达率为60.0%，低于原发灶中的90.9%^[18]^。这些结果提示，尽管多数标志物在转移灶中保持了其基本的表达谱，但其阳性率可能受肿瘤异质性、样本类型或处理方式的影响而有所波动。同时，单个标志物在不同亚型间存在表达重叠，限制了其独立用于分型的特异性。本研究中神经内分泌标志物Syn与CD56在小细胞癌中分别呈87.0%和81.8%的阳性表达，但在肺腺癌中分别有6.8%和15.2%的表达，这与既往报道的10%-30%^[19]^表达率相符。P63虽在肺鳞癌中阳性率为73.7%，但在肺腺癌中也有7.8%的阳性表达，也与之前文献结论一致^[20]^。这些交叉表达现象提示，依赖单一标志物易导致分型误判。因此，必须采用组合抗体进行检测与综合判读，克服其局限性。本研究中，腺癌的诊断依赖TTF-1/Napsin A双阳性模式，并结合鳞状上皮癌与神经内分泌标志物的阴性结果：鳞癌依赖P40/P63的表达组合；小细胞癌依赖Syn/CD56等神经内分泌标志物的强阳性。本研究结果符合TIS系统所倡导的整合诊断理念，联合应用一组互补的ICC标志物，可以对形态学初诊进行验证与补充。

本研究存在一定局限性。首先，作为一项回顾性研究，所有纳入病例均为经组织病理学和/或临床病史确诊的肺癌患者，缺乏非肿瘤性浆膜腔积液作为对照。虽然确保了研究队列的同质性，有利于评估TIS系统在肺癌人群中的诊断与分型效能，但无法全面反映该系统对浆膜腔积液性质的鉴别诊断性能。其次，研究样本均来源于单中心肿瘤专科医院，可能存在选择性偏倚，研究结论的外推性需要进一步验证。未来研究应该纳入包含良性与恶性积液的对照队列，并在多中心、不同层级医疗机构中开展前瞻性验证，以全面评估TIS系统在浆膜腔积液诊断和性质鉴别中的临床应用价值。此外，本研究主要聚焦于形态学与ICC检测，未系统纳入分子检测数据分析。在精准医学发展的推动下，基于浆膜腔积液的分子检测已经逐步成为肺癌个体化治疗的关键环节，后续研究将进一步探讨细胞病理学分级与分子特征之间的关联，及其在指导临床治疗决策中的价值。未来，随着“形态-蛋白-基因”整合诊断模式日益成熟，TIS系统的临床应用将更加广阔。

综上所述，遵循TIS系统构建的“形态学初诊，辅助技术确证”分层诊断路径，在提升诊断准确性、优化不确定性病例分流、实现恶性浆膜腔积液的分型与溯源等方面，展示出显著的临床应用价值。本研究基于肿瘤专科医院的大样本数据，验证了该系统在肺癌相关浆膜腔积液中的诊断效能。值得注意的是，TIS系统的核心价值不仅在于提升单一中心的诊断水平，更在于推动不同层级医疗机构之间细胞病理学诊断的标准化与同质化。因此，建议未来从以下方面推进相关工作：（1）在基层或综合医院开展多中心验证研究，进一步评估该系统在资源有限条件下的适用性与可推广性；（2）在此基础上，牵头制定基于TIS系统的适合国情的《肺癌浆膜腔积液细胞病理学诊断规范化专家共识》；（3）建立针对肺癌不同亚型的浆膜腔积液ICC标志物组合推荐方案，为临床提供明确的抗体选择与判读标准。通过这些具体举措，可为全国范围内浆膜腔积液细胞病理学诊断的规范化建设提供更为坚实的循证基础，更好地服务于肿瘤精准医疗。
